# Natural language processing in the era of large language models

**DOI:** 10.3389/frai.2023.1350306

**Published:** 2024-01-12

**Authors:** Arkaitz Zubiaga

**Affiliations:** School of Electronic Engineering and Computer Science, Queen Mary University of London, London, United Kingdom

**Keywords:** natural language processing, large language models (LLM), language models (LMs), specialty grand challenge, generative AI

## 1 Overview

Since their inception in the 1980s, language models (LMs) have been around for more than four decades as a means for statistically modeling the properties observed from natural language (Rosenfeld, 2000). Given a collection of texts as input, a language model computes statistical properties of language from those texts, such as frequencies and probabilities of words and surrounding context, which can then be used for different purposes including natural language understanding (NLU), generation (NLG), reasoning (NLR) and, more broadly, processing (NLP) (Dong et al., 2019). Such statistical approach to modeling natural language has sparked debate for decades between those who argue that language can be modeled through the observation and probabilistic representation of patterns, and those who argue that such an approach is rudimentary and that proper understanding of language needs grounding in linguistic theories (Mitchell and Krakauer, 2023).

It has only been recently that, as a consequence of the increase in the availability of text collections and in the access to improved computational resources, large language models (LLMs) have been introduced in the scientific community by revolutionizing the NLP field (Min et al., 2023). Following the same foundational intuition as traditional LMs introduced in the 1980s, LLMs scale up the statistical language properties garnered from large text collections. Following the same logic of modeling statistical properties of languages as traditional LMs, researchers have demonstrated that, with today's computational resources, it is possible to train much larger LLMs which are trained from huge collections of text that on occasions can even include almost the entire Web. This is however not without controversy, not least because use of such large-scale collections of text prioritizes quantity over quality (Li et al., 2023a), as indeed one loses control of what data is being fed into the model when the whole Web is being used, which in addition to valuable information contains offensive content and misinformation (Derczynski et al., 2014; Cinelli et al., 2021; Yin and Zubiaga, 2021).

The surge of LLMs has been incremental since the late 2010s and has come in waves. Following a wave that introduced word embedding models such as word2vec (Mikolov et al., 2013) and GloVe (Pennington et al., 2014) for compact representation of words in the form of embeddings, the first major wave came with the emergence of LLMs built on top of the Transformer architecture (Vaswani et al., 2017), including BERT (Devlin et al., 2019), RoBERTa (Liu et al., 2019) and T5 (Raffel et al., 2020). A more recent wave has led to a surge of models for generative AI including chatbots like ChatGPT, Google Bard, as well as open source alternatives such as LLaMa (Touvron et al., 2023), Alpaca (Taori et al., 2023) and Lemur (Xu et al., 2023). These have in turn motivated the creation of different ways of leveraging these LLMs, including through prompting methods (Liu et al., 2023) such as Pattern Exploiting Training (PET) (Schick and Schütze, 2021) for few-shot text classification as well as methods for NLG (Sarsa et al., 2022). An LLM is typically a model which is pre-trained on existing large-scale datasets, which involves significant computational power and time, whereas these models can later be fine-tuned to specific domains with less effort (Bakker et al., 2022).

In recent years, LLMs have demonstrated to achieve state-of-the-art performance across many NLP tasks, having in turn become the *de facto* baseline models to be used in many experimental settings (Mars, 2022). There is however evidence that the power of LLMs can also be leveraged for malicious purposes, including the use of LLMs to assist with completion of school assignments by cheating (Cotton et al., 2023), or to generate content that is offensive or spreads misinformation (Weidinger et al., 2022).

The great performance of LLMs has also inevitably provoked some fear in society that artificial intelligence tools may eventually take up many people's jobs (George et al., 2023), hence questioning the ethical implications they may have on society. This has in turn sparked research, with recent studies suggesting to embrace AI tools as they can in fact support and boost the performance of, rather than replace, human labor (Noy and Zhang, 2023).

## 2 Limitations and open challenges

The success of LLMs is not without controversy, which is in turn shaping up ongoing research in NLP and opening up avenues for more research in improving these LLMs. The following are some of the key limitations of LLMs which need further exploration.

### 2.1 Black box models

After the release of the first major LLM-based chatbot system that garnered mainstream popularity, OpenAI's ChatGPT, concerns emerged around the black box nature of the system. Indeed, there is no publicly available information on how ChatGPT was implemented as well as what data they used for training their model. From the perspective of NLP researchers, this raises serious concerns about the transparency and reproducibility of such model, not only because one does not know what is going on in the model, but also because it hinders reproducibility (Belz et al., 2021). If one runs some experiments using ChatGPT on a particular date, there is no guarantee that somebody else can reproduce those results at a later date (or, arguably, even on the same date), which reduces the validity and potential for impact and generalisability of ChatGPT-based research.

To mitigate the impact, and increase our understanding, of black box models like ChatGPT, researchers have started investigating methods for reverse engineering those models, for example by trying to find out what data a model may have used for training (Shi et al., 2023).

Luckily, however, there is a recent surge of open source models in the NLP scientific community, which have led to the release of models like Facebook's LLaMa 2 (Touvron et al., 2023) and Stanford's Alpaca (Taori et al., 2023), as well as multilingual models like BLOOM (Scao et al., 2023). Recent studies have also shown that the performance of these open source alternatives is often on par with closed models like ChatGPT (Chen et al., 2023).

### 2.2 Risk of data contamination

Data contamination occurs when “downstream test sets find their way into the pretrain corpus” (Magar and Schwartz, 2022). Where an LLM trained on large collections of text has already seen the data it is then given at test time for evaluation, the model will then exhibit an impressive yet unrealistic performance score. Research has in fact shown that data contamination can be frequent and have a significant impact (Deng et al., 2023; Golchin and Surdeanu, 2023). It is therefore crucial that researchers ensure that the test data has not been seen by an LLM before, for a fair and realistic evaluation. This is however challenging, if not nearly impossible, to figure out with black box models, which again encourages the use of open source, transparent LLMs.

### 2.3 Bias in LLM models

The use of large-scale datasets for training LLMs also means that those datasets are very likely to contain biased or stereotyped information, which has been shown that LLMs amplify (Gallegos et al., 2023; Li et al., 2023b). Research has shown that text generated by LLMs includes stereotypes against women when writing reference letters (Wan et al., 2023), suggesting that LLMs in fact amplify gender biases inherent in the training data leading to an increased probability of stereotypical linking between gender groups and professions (Kotek et al., 2023). Another recent study (Navigli et al., 2023) has also shown that LLMs exhibit biases against numerous demographic characteristics, including gender, age, sexual orientation, physical appearance, disability or race, among others.

### 2.4 Generation of offensive content

Biases inherent in LLMs are at times exacerbated to even generate content that can be deemed offensive (Weidinger et al., 2021). Research in this direction is looking at how to best curate the training data fed to LLMs to avoid learning offensive samples, as well as in eliciting generation of those harmful texts to understand their origin (Srivastava et al., 2023). This research is highly linked with the point above on bias and fairness in LLMs, and therefore both could be studied jointly by looking at the reduction of biases and harm.

Some systems, such as OpenAI's ChatGPT, acknowledge the risk of producing offensive content in their terms of service^1^:

“Our Services may provide incomplete, incorrect, or offensive Output that does not represent OpenAIs views. If Output references any third party products or services, it doesnt mean the third party endorses or is affiliated with OpenAI.”

### 2.5 Privacy

LLMs can also capture sensitive information retrieved from its training data. While this information is encoded in embeddings which are not human readable, it has been found (Pan et al., 2020) that an adversarial user can reverse engineer those embeddings to recover the sensitive information, which can have damaging consequences for the relevant individuals. While research investigating these vulnerabilities of LLMs is still in its infancy, there is awareness of the urgency of such research to make LLMs robust to privacy attacks (Guo et al., 2022; Rigaki and Garcia, 2023; Shayegani et al., 2023).

### 2.6 Imperfect accuracy

Despite initial impressions that LLMs achieve an impressive performance, a closer look and investigation into model outputs shows that there is significant room for improvement. Evaluation of LLMs has in turn become a fertile area of research (Chang et al., 2023).

Aware of the many shortcomings and inaccurate outputs of LLMs, companies responsible for the production and publication of major LLMs all have disclaimers about the limitations of their models. For example, ChatGPT owner OpenAI acknowledges that:

“Output may not always be accurate. You should not rely on Output from our Services as a sole source of truth or factual information, or as a substitute for professional advice.”

Google also warns^2^ about the limitations of its LLM-based chatbot Bard, as follows:

“Bard is an experimental technology and may sometimes give inaccurate or inappropriate information that doesnt represent Googles views.”

“Dont rely on Bards responses as medical, legal, financial, or other professional advice.”

Facebook also has a similar disclaimer^3^ for its flagship model LLaMa 2:

“Llama 2s potential outputs cannot be predicted in advance, and the model may in some instances produce inaccurate, biased or other objectionable responses to user prompts. Therefore, before deploying any applications of Llama 2, developers should perform safety testing and tuning tailored to their specific applications of the model.”

### 2.7 Model hallucination

Responses and outputs generated by LLMs often deviate from common sense, where for example a generated text can start discussing a particular topic, then shifting to another unrelated topic which is not intuitive, or even stating wrong facts. LLM hallucination has been defined as “the generation of content that deviates from the real facts, resulting in unfaithful outputs” (Maynez et al., 2020; Rawte et al., 2023). Efforts toward better understanding model hallucination is focusing on different tasks, including detection, explanation, and mitigation (Alkaissi and McFarlane, 2023; Zhang et al., 2023), with some initial solutions proposed to date, such as Retrieval-Augmented Generation (RAG) (Lewis et al., 2020).

### 2.8 Lack of explainability

The complexity of LLM models means that it is often very difficult to understand why it makes certain predictions or produces certain outputs. This also means that it is very difficult to provide explanations on model outputs to system users, which calls for more investigation into furthering the explainability of LLMs (Danilevsky et al., 2020; Gurrapu et al., 2023; Zhao et al., 2023).

## 3 Concluding remarks

The introduction and surge in popularity of LLMs has impacted and reshaped NLP research. Much of the NLP research and methods slightly over a decade ago focused on the representation of words using bag-of-words and TF-IDF based methods and the use of machine learning algorithms such as Logistic Regression or Support Vector Machine classifiers. The increase in computational capacity to handle large-scale datasets and for more complex computing has led to the renaissance of deep learning models and in turn the emergence of LLMs. The latter have shown to achieve unprecedented performance across a range of downstream NLP tasks, but have also opened up numerous avenues for future research aiming to tackle the limitations and weaknesses of LLMs. Much of this research will need to deal with the better curation of the data fed to train LLMs, which in the current circumstances has shown to have severe risks in aspects such as fairness, privacy and harm.

## Author contributions

AZ: Writing – original draft, Writing – review & editing.

## Footnotes

^1^ https://openai.com/policies/terms-of-use

^2^ https://support.google.com/bard/answer/13594961?hl=en

^3^ https://github.com/facebookresearch/llama/blob/main/MODEL_CARD.md

## References

[B1] Alkaissi H. McFarlane S. I. (2023). Artificial hallucinations in chatgpt: implications in scientific writing. Cureus 15, 2. 10.7759/cureus.35179 36811129 PMC9939079

[B2] Bakker M. Chadwick M. Sheahan H. Tessler M. Campbell-Gillingham L. Balaguer J.. (2022). Fine-tuning language models to find agreement among humans with diverse preferences. Adv. Neural Inform. Proc. Syst. 35, 38176–38189.

[B3] Belz A. Agarwal S. Shimorina A. Reiter E. (2021). “A systematic review of reproducibility research in natural language processing,” in Proceedings of the 16th Conference of the European Chapter of the Association for Computational Linguistics: Main Volume. Kerrville, TX: Association for Computational Linguistics, 381–393.

[B4] Chen H. Jiao F. Li X. Qin C. Ravaut M. Zhao R.. (2023). Chatgpt's one-year anniversary: are open-source large language models catching up? arXiv. 10.48550/arXiv.2311.16989

[B5] Chang Y. Wang X. Wang J. Wu Y. Zhu K. Chen H.. (2023). A survey on evaluation of large language models. arXiv. 10.48550/arXiv.2307.03109

[B6] Cinelli M. Pelicon A. Mozetič I. Quattrociocchi W. Novak P. K. Zollo F. (2021). Dynamics of online hate and misinformation. Scient.Rep. 11, 22083. 10.1038/s41598-021-01487-w 34764344 PMC8585974

[B7] Cotton D. R. Cotton P. A. Shipway J. R. (2023). “Chatting and cheating: Ensuring academic integrity in the era of chatgpt,” in Innovations in Education and Teaching International (Oxfordshire: Routledge), 1–12.

[B8] Danilevsky M. Qian K. Aharonov R. Katsis Y. Kawas B. Sen P. (2020). “A survey of the state of explainable ai for natural language processing,” in Proceedings of the 1st Conference of the Asia-Pacific Chapter of the Association for Computational Linguistics and the 10th International Joint Conference on Natural Language Processing (Association for Computational Linguistics), 447–459.

[B9] Deng C. Zhao Y. Tang X. Gerstein M. Cohan A. (2023). Investigating data contamination in modern benchmarks for large language models. arXiv. 10.48550/arXiv.2311.09783

[B10] Derczynski L. Bontcheva K. Lukasik M. Declerck T. Scharl A. Georgiev G.. (2014). “Pheme: computing veracity: the fourth challenge of big social data,” in Proceedings of ESWC EU Project Networking (Vienna: Semantic Technology Institute International).

[B11] Devlin J. Chang M.-W. Lee K. Toutanova K. (2019). “Bert: Pre-training of deep bidirectional transformers for language understanding,” in Proceedings of the 2019 Conference of the North American Chapter of the Association for Computational Linguistics: Human Language Technologies, Volume 1 (Long and Short Papers), Kerrville, TX: Association for Computational Linguistics, 4171–4186.

[B12] Dong L. Yang N. Wang W. Wei F. Liu X. Wang Y.. (2019). “Unified language model pre-training for natural language understanding and generation,” in Advances in Neural Information Processing Systems (Red Hook, NY: Curran Associates, Inc.), 32.

[B13] Gallegos I. O. Rossi R. A. Barrow J. Tanjim M. M. Kim S. Dernoncourt F.. (2023). Bias and fairness in large language models: a survey. arXiv. 10.48550/arXiv.2309.00770

[B14] George A. S. George A. H. Martin A. G. (2023). Chatgpt and the future of work: a comprehensive analysis of ai's impact on jobs and employment. Partners Universal Int. Innovat. J. 1, 154–186.

[B15] Golchin S. Surdeanu M. (2023). Time travel in llms: tracing data contamination in large language models. arXiv. 10.48550/arXiv.2308.08493

[B16] Guo S. Xie C. Li J. Lyu L. Zhang T. (2022). Threats to pre-trained language models: Survey and taxonomy. arXiv. 10.48550/arXiv.2202.06862

[B17] Gurrapu S. Kulkarni A. Huang L. Lourentzou I. Batarseh F. A. (2023). Rationalization for explainable nlp: a survey. Front. Artif. Intellig. 6, 1225093. 10.3389/frai.2023.1225093 37818431 PMC10560994

[B18] Kotek H. Dockum R. Sun D. (2023). “Gender bias and stereotypes in large language models,” in Proceedings of The ACM Collective Intelligence Conference (New York, NY: Association for Computing Machinery), 12–24.

[B19] Li M. Zhang Y. Li Z. Chen J. Chen L. Cheng N.. (2023a). From quantity to quality: boosting llm performance with self-guided data selection for instruction tuning. arXiv. 10.48550/arXiv.2308.12032

[B20] Li Y. Du M. Song R. Wang X. Wang Y. (2023b). A survey on fairness in large language models. arXiv. 10.48550/arXiv.2308.10149

[B21] Lewis P. Perez E. Piktus A. Petroni F. Karpukhin V. Goyal N.. (2020). Retrieval-augmented generation for knowledge-intensive nlp tasks. Adv. Neural Inform. Proc.Syst. 33, 9459–9474.

[B22] Liu Y. Ott M. Goyal N. Du J. Joshi M. Chen D.. (2019). Roberta: a robustly optimized bert pretraining approach. arXiv. 10.48550/arXiv.1907.11692

[B23] Liu P. Yuan W. Fu J. Jiang Z. Hayashi H. Neubig G. (2023). Pre-train, prompt, and predict: a systematic survey of prompting methods in natural language processing. ACM Comput. Surv. 55, 1–35. 10.1145/3560815

[B24] Magar I. Schwartz R. (2022). “Data contamination: From memorization to exploitation,” in Proceedings of the 60th Annual Meeting of the Association for Computational Linguistics (Volume 2: Short Papers) Kerrville, TX: Association for Computational Linguistics, 157–165.

[B25] Mars M. (2022). From word embeddings to pre-trained language models: a state-of-the-art walkthrough. Appl. Sci. 12, 8805. 10.3390/app12178805

[B26] Maynez J. Narayan S. Bohnet B. McDonald R. (2020). “On faithfulness and factuality in abstractive summarization,” in Proceedings of the 58th Annual Meeting of the Association for Computational Linguistics. Kerrville, TX: Association for Computational Linguistics, 1906.

[B27] Mikolov T. Chen K. Corrado G. Dean J. (2013). Efficient estimation of word representations in vector space. arXiv. 10.48550/arXiv.1301.3781

[B28] Min B. Ross H. Sulem E. Veyseh A. P. B. Nguyen T. H. Sainz O.. (2023). Recent advances in natural language processing via large pre-trained language models: a survey. ACM Computing Surveys 56, 1–40. 10.1145/3605943

[B29] Mitchell M. Krakauer D. C. (2023). The debate over understanding in ais large language models. Proc. National Acad. Sci. 120, e2215907120. 10.1073/pnas.2215907120 36943882 PMC10068812

[B30] Navigli R. Conia S. Ross B. (2023). Biases in large language models: origins, inventory and discussion. ACM J. Data Inform. Qual. 15, 1–21. 10.1145/3597307

[B31] Noy S. Zhang W. (2023). Experimental Evidence on the Productivity Effects of Generative Artificial Intelligence. Amsterdam: Elsevier Inc. 10.1126/science.adh2586 37440646

[B32] Pan X. Zhang M. Ji S. Yang M. (2020). “Privacy risks of general-purpose language models,” in 2020 IEEE Symposium on Security and Privacy (SP). San Francisco, CA: IEEE, 1314-1331.

[B33] Pennington J. Socher R. Manning C. D. (2014). “Glove: Global vectors for word representation,” in Proceedings of the 2014 Conference on Empirical Methods in Natural Language Processing (EMNLP). Stanford, CA: Stanford University, 1532–1543.

[B34] Raffel C. Shazeer N. Roberts A. Lee K. Narang S. Matena M.. (2020). Exploring the limits of transfer learning with a unified text-to-text transformer. J. Mach. Learn. Res. 21, 5485–5551.

[B35] Rawte V. Chakraborty S. Pathak A. Sarkar A. Tonmoy S. Chadha A.. (2023). The troubling emergence of hallucination in large language models-an extensive definition, quantification, and prescriptive remediations. arXiv. 10.18653/v1/2023.emnlp-main.155

[B36] Rigaki M. Garcia S. (2023). A survey of privacy attacks in machine learning. ACM Comp. Surv. 56, 1–34. 10.1145/3624010

[B37] Rosenfeld R. (2000). Two decades of statistical language modeling: where do we go from here? Proc. IEEE 88, 1270–1278. 10.1109/5.880083

[B38] Sarsa S. Denny P. Hellas A. Leinonen J. (2022). “Automatic generation of programming exercises and code explanations using large language models,” in Proceedings of the 2022 ACM Conference on International Computing Education Research (New York, NY: Association for Computing Machinery), 27–43. 10.1145/3501385.3543957

[B39] Scao T. L. Fan A. Akiki C. Pavlick E. Ili S. Hesslow D.. (2023). Bloom: A 176b-parameter open-access multilingual language model. arXiv. 10.48550/arXiv.2211.05100

[B40] Schick T. Schütze H. (2021). “Exploiting cloze-questions for few-shot text classification and natural language inference,” in Proceedings of the 16th Conference of the European Chapter of the Association for Computational Linguistics: Main Volume (Association for Computational Linguistics), 255–269.

[B41] Shi W. Ajith A. Xia M. Huang Y. Liu D. Blevins T.. (2023). Detecting pretraining data from large language models. arXiv. 10.48550/arXiv.2310.16789

[B42] Shayegani E. Mamun M. A. A. Fu Y. Zaree P. Dong Y. Abu-Ghazaleh N. (2023). Survey of vulnerabilities in large language models revealed by adversarial attacks. arXiv. 10.48550/arXiv.2310.10844

[B43] Srivastava A. Ahuja R. Mukku R. (2023). No offense taken: eliciting offensiveness from language models. arXiv. 10.48550/arXiv.2310.00892

[B44] Taori R. Gulrajani I. Zhang T. Dubois Y. Li X. Guestrin C.. (2023). Stanford Alpaca: An Instruction-Following Llama Model. Available online at: https://github.com/tatsu-lab/stanford_alpaca (accessed December 1, 2023).

[B45] Touvron H. Lavril T. Izacard G. Martinet X. Lachaux M.-A. Lacroix T.. (2023). Llama: Open and efficient foundation language models. arXiv. 10.48550/arXiv.2302.13971

[B46] Vaswani A. Shazeer N. Parmar N. Uszkoreit J. Jones L. Gomez A. N.. (2017). “Attention is all you need,” in Advances in Neural Information Processing Systems (Red Hook, NY: Curran Associates, Inc.), 30.

[B47] Wan Y. Pu G. Sun J. Garimella A. Chang K.-W. Peng N. (2023). “ kelly is a warm person, joseph is a role model”: Gender biases in llm-generated reference letters. arXiv. 10.18653/v1/2023.findings-emnlp.243

[B48] Weidinger L. Uesato J. Rauh M. Griffin C. Huang P.-S. Mellor J.. (2022). “Taxonomy of risks posed by language models,” in Proceedings of the 2022 *ACM Conference on Fairness, Accountability, and Transparency*. New York: Association for Computing Machinery, 214–229. 10.1145/3531146.3533088

[B49] Weidinger L. Mellor J. Rauh M. Griffin C. Uesato J. Huang P.-S.. (2021). Ethical and social risks of harm from language models. arXiv. 10.48550/arXiv.2112.04359

[B50] Xu Y. Su H. Xing C. Mi B. Liu Q. Shi W.. (2023). Lemur: Harmonizing natural language and code for language agents. arXiv. 10.48550/arXiv.2310.06830

[B51] Yin W. Zubiaga A. (2021). Towards generalisable hate speech detection: a review on obstacles and solutions. PeerJ Comp. Sci. 7, e598. 10.7717/peerj-cs.598 34239978 PMC8237316

[B52] Zhang Y. Li Y. Cui L. Cai D. Liu L. Fu T.. (2023). Siren's song in the ai ocean: a survey on hallucination in large language models. arXiv. 10.48550/arXiv.2309.01219

[B53] Zhao H. Chen H. Yang F. Liu N. Deng H. Cai H.. (2023). Explainability for large language models: a survey. arXiv. 10.1145/3639372

